# A Surface Modified Carbon Nanotube Fiber as a Microelectrode for the pH-Dependent Electrochemical Detection of Vitamin C

**DOI:** 10.1021/acsomega.5c05410

**Published:** 2025-09-01

**Authors:** Warda Mansur, Rizwan Shoukat, Murat Kaleli, Salih Akyürekli, Muhammad Ahmad, Amel Y. Ahmed, Abid Ali

**Affiliations:** † Department of Chemistry, 117305 The University of Lahore, 1-Km Defence Road, Lahore 54590, Pakistan; ‡ Department of Mechanical, Chemical and Materials Engineering, University of Cagliari, via Marengo 2, 09123, Cagliari, Sardegna 09124, Italy; § Innovative Technologies Application and Research Center (YETEM), 52994 Süleyman Demirel University, Isparta 32260, Turkiye; ∥ University of Education Lahore, College Road, Johar Town, Lahore 54770, Pakistan; ⊥ Department of Chemistry, Faculty of Science, 114800 King Faisal University, Al-Ahsa 31982, Saudi Arabia

## Abstract

A pH-dependent electrochemical sensor based on nickel–cobalt oxide modified carbon nanotube fiber (NiCo–O@CNTF) has been fabricated via a simple electrochemical approach and utilized in the nonenzymatic detection of ascorbic acid (AA) with varying pH. Scanning electron microscopy (SEM), energy-dispersive electron microscopy (EDX), Raman, and FTIR were utilized to investigate the structural morphologies, elemental mapping, and electronic vibrational modes within the modified fiber (NiCo–O@CNTF) and revealed the successful fabrication of the electrode. Electrochemical characterization, including cyclic voltammetry and chronoamperometry, exhibited an enhanced anodic response in buffer solutions (varying pH), achieving a high sensitivity of 62.39 μA cm^–2^ mM^–1^, a low detection limit of 16 μM, and a rapid response time of 5 s at pH 8.0. These results highlight NiCo–O@CNTF as a flexible and disposable microelectrode platform for wearable AA sensing applications.

## Graphical abstract

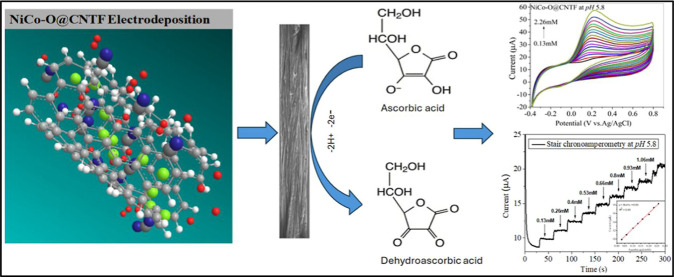

## 1 Introduction

Ascorbic acid (AA), also known as vitamin C (C_6_H_8_O_6_), is a hydro-soluble, essential organic compound crucial for human beings, as the body cannot synthesize it on its own, and it must be obtained from external sources.^1–3^ Usually, the reference range of AA in the body is 0.6–2 mg/dL, and its deficiency can cause several disorders, such as scurvy and anemia, while excessive consumption may lead to adverse consequences, including diarrhea, kidney stones, and digestive disorders.^4–8^ The primary role of vitamin C is to preserve shelf life by maintaining odor, color, taste, and stability, while its singlet oxygen scavenging mechanism protects oxidizable components, assisting in quality control.^9–11^ Its significance in antioxidant defense, cholesterol metabolism, and collagen formation makes precise measurements critical to well-being.^12–14^ Analytical techniques, including HPLC, colorimetric methods, and various electrochemical approaches, have been employed for the monitoring of AA.^12,15,16^ Among these, electrochemical methods are favored for their cost-effectiveness, high selectivity, sensitivity, ease of use, and rapid analysis.^17^ In electrochemical detection, sensors vary widely from enzymatic to nonenzymatic routes.^18^ While enzymatic sensors have received much attention, along with various limitations, such as complex immobilization and susceptibility to pH, humidity, and temperature, which restrict their frequent usage.^19,20^ Therefore, nonenzymatic sensors are preferred due to their increased stability and endurance for the sensitive and selective detection of analytes.^21–23^ Based on the resources, transition metal oxides (TMOs) provide a unique platform for biosensing applications due to their distinct electronic configurations.^24^ Oxides, specifically NiO and Co_3_O_4_, emerged as an alternative electrode material and are extensively used as pseudocapacitive electrodes due to their facile synthesis, cost-effectiveness, and thermal and chemical durability.^25–27^ For instance, a combination of such oxides as discussed above can significantly enhance capacitive performance and improve overall efficiency.^28,29^

Carbon-based materials such as carbon nanotubes, graphene, activated carbon, and metal oxides are widely used as electrode materials.^30^ CNTFs have been utilized in multiple miniaturized devices and are a suitable alternative to expensive Pt-based materials due to their excellent electrical properties, high mechanical strength, and increased stability.^31–34^ Furthermore, this method has been widely used in recent years to produce skin-adhered electrodes for microanalytical devices since it does not require expensive equipment.^35^

Researchers found that current methodologies for detecting ascorbic acid encounter challenges, including signal interference from other chemical components as well as costly and complex sample preparation. Conventional chemical techniques, such as spectrophotometry and chromatography, are sensitive and specific but demand expensive equipment, trained workers, and substantial preparation.^36,37^ Thus, a more accurate, economical, and efficient approach is required to overcome such limitations by providing high sensitivity and selectivity at a low cost with a rapid response time.^38^

Advanced nanotechnology led to the development of various nanomaterials and complex sensors.^39^ Numerous biosensors have tremendous potential for continuous, noninvasive monitoring of ascorbic acid (AA) levels in body fluids such as sweat and tears.^40^ pH sensors are also critical for monitoring chemical, biological, and medicinal applications.^41,42^ The sensing capabilities at various pH levels indicate a linear relationship between applied potential and electric current.^43^ However, variations in the pH of the solution may impact the sensor due to enzyme activity.^44^ Since the oxidation of ascorbic acid (AA) is extremely pH-dependent, body fluid pH changes with physiological events. AA-sensing electrodes must be integrated with a pH adjustment system to ensure precise and reliable detection.^45^ Hence, flexible microelectrodes have recently gained considerable attention for wearable and point-of-care electrochemical sensing due to their lightweight, adaptable, and integrative properties.^46^ Compared to rigid electrodes, fiber-based flexible microelectrodes offer a high surface-to-volume ratio, excellent mechanical stability, and facile integration with textile or skin-mounted platforms.^47^ Carbon nanotube fibers (CNTFs) provide a conductive and flexible framework that can be easily modified with catalytic nanomaterials, enabling efficient electron transfer and enhanced sensitivity.^48^ Such features make CNTF-based flexible microelectrodes highly suitable for real-time monitoring of biomolecules, positioning them as promising candidates for next-generation wearable sensors.^49,50^

In this study, an electrodeposition method was employed to modify carbon nanotube fiber with bimetallic nickel and cobalt oxide, yielding a NiCo–O@CNTF modified electrode with boosted electrocatalytic activity toward electrochemical sensing of ascorbic acid. Material was subsequently developed into a low-cost, nonenzymatic electrochemical biosensor for ascorbic acid, which is significantly influenced by pH and affects its sensitivity, selectivity, and stability. Cyclic voltammetry and chronoamperometry were used to examine the electrochemical behavior of the fabricated electrode in 0.1 M phosphate buffer at varying pH levels. The anodic peak at pH 8.0 showed a greater rise in current with a higher sensitivity of 62.39 μA cm^–2^ mM^–1^ and a lower detection limit of 16 μM, in contrast to that at pH 5.8. These findings highlight the significant changes in ascorbic acid’s redox behavior and electrochemical kinetics under alkaline conditions, emphasizing its pH sensitivity and impact on analytical applications and stability across various pH levels.

## 2 Experimental Section

### 2.1 Materials

#### 2.1.1 Fabrication of CNT Fiber-Shaped Electrodes

CNT fibers were fabricated via wet chemical methods^51^ and used as received. Phosphate buffer salt (monobasic KH_2_PO_4_) and (dibasic K_2_HPO_4_) and ethanol (C_2_H_5_OH) were obtained from Merck. Ascorbic acid, sulfuric acid (H_2_SO_4_), nickel chloride hexahydrate (NiCl_2_·6H_2_O), and cobalt chloride hexahydrate (CoCl_2_·6H_2_O) were acquired from Sigma-Aldrich. Indium metal, hydrochloric acid (HCl), nitric acid (HNO_3_), and distilled water were obtained from the laboratory’s plant. The acquired chemicals, termed analytical standards, were used without any further purification. For functionalization, a CNT fiber of ∼150 μm diameter and ∼2 cm length was immersed in 0.1 M sulfuric acid (H_2_SO_4_) for several minutes to functionalize its surface. The fiber was washed and rinsed with ethanol and distilled water and oven-dried at 60 °C. The glass slide acted as the support material with the carbon nanotube fiber attached via indium metal at one end and Teflon tape at the other. The NiCo–O@CNTF electrode was prepared by the electrodeposition of nickel and cobalt precursors onto a functionalized carbon nanotube fiber. The deposition electrolyte consisted of an equimolar (0.1 M) aqueous solution of NiCl_2_·6H_2_O and CoCl_2_·6H_2_O. Chronoamperometry methods were performed at the applied potential of −1.1 V for 600 s under constant stirring while a CNT fiber was used as the working electrode (Scheme 1). Following deposition, the electrode was washed thoroughly and dried in an oven at 120 °C for 24 h and allowed to cool down prior to electrochemical analysis.

**Scheme 1:**
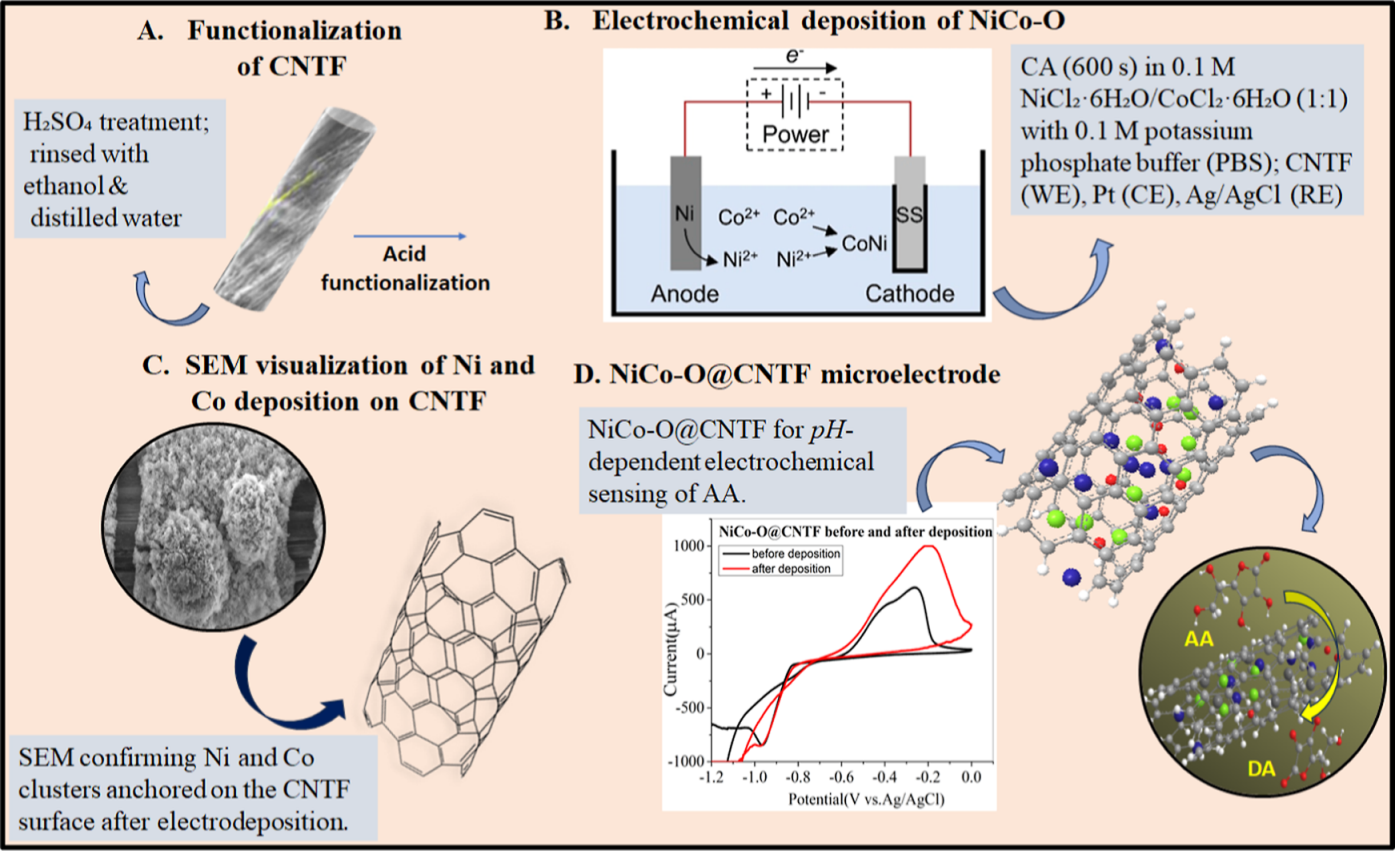
Schematic illustration of electrodeposition of NiCo–O over the CNTs-based fiber electrode toward the detection of ascorbic acid

### 2.2 Materials Characterization

Surface morphologies and elemental composition of the CNT fiber materials were analyzed via SEM (FEI Quanta FEG 250 EDAX/EDS). Raman (WITec alpha 300R) and FTIR (JASCO FT/IR 4700) have been employed for the material composition and identity of the different vibrational modes associated with the CNTs and metal-based oxides. All the electrochemical studies were carried out using a Gamry Interface 1010E Potentiostat.

### 2.3 Electrochemical Studies

Voltammetric measurements were performed using a three-electrode setup with a potentiostat/galvanostat (Gamry Interface 1010E). A platinum wire was employed as the counter electrode, while CNT fibers and silver/silver chloride were used as the Ag/AgCl working and reference electrodes. The electrolyte used was phosphate buffer solution (PBS) at different pH. All measurements were repeated three times at room temperature (25 °C).

## 3 Results and Discussion

### 3.1 Material Characterization

#### 3.1.1 Scanning Electron Microscopy

Scanning electron microscopy (SEM) was employed to examine the morphology of the carbon nanotube fiber. SEM images in Figure 1a,b displayed aligned twisted bundles of nanotubes, revealing their distinctive fibrous and entangled network structure. Additionally, SEM also highlighted the fiber’s density and homogeneity, which affect its mechanical strength, electrical conductivity, and functionality in devices like electrodes or sensors. The aligned structure of the spinnable CNTs is responsible for enhancing electrical and optical properties, with boosted mechanical strength of this type of fiber. CNTFs are composed of coiled nanotubes and exhibit exceptional mechanical, thermal, and electrical conductivities above 10^4^ S·m^–1^, which makes them ideal for complex fiber electronic devices for various applications.^52–54^

**Figure 1:**
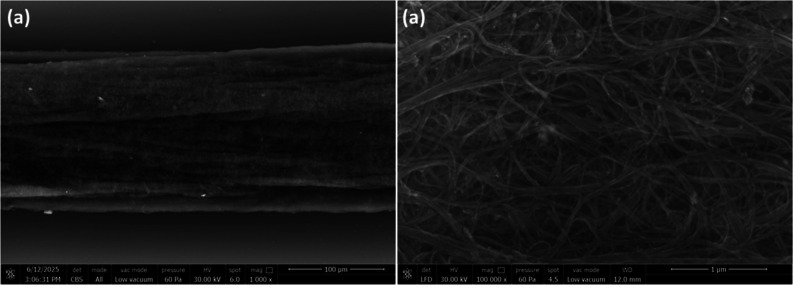
SEM images of pristine CNT fiber with (a) lower and (b) higher resolution.

The modified electrode NiCo–O@CNTF was taken at various magnifications (200–10 μm), confirming the efficient electrodeposition of nickel–cobalt oxide nanoparticles on CNT fiber, as shown in Figure 2a–d. SEM reveals that metal characteristics influence the density of nanomaterials in terms of per unit area, growth rate, and particle size, leading to distinct variations in the nanoparticle dispersion across the CNT surface. The micrograph of this complex lattice, with a broad surface and a narrow pore size distribution, reveals bimetallic electrode particle clusters of various sizes at lower resolutions and sphere-like structures observed at greater resolution. The wide surface area optimizes electrode–electrolyte interactions, while the appropriate pore size promotes ion transport and redox reactions during charge transfer.

**Figure 2:**
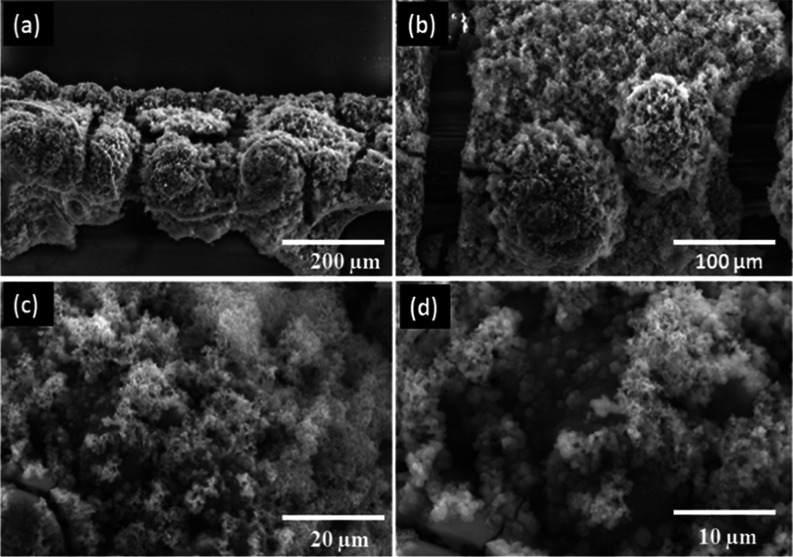
SEM images of modified CNTs fiber (NiCo–O@CNTF) at (a,b) lower and (c,d) higher resolutions.

#### 3.1.2 EDS Analysis

Energy-dispersive X-ray spectroscopy (EDS) has enabled element mapping and their abundance ratios at the nanoscale and even at atomic column resolution, to analyze the composition of the sample.^55^ EDS is a solid analytical technique used to determine the elemental composition and distribution of materials. Coupled with scanning electron microscopy, it provides qualitative and semiquantitative information by detecting characteristic X-ray emissions from the specimen under electron beam excitation. In this work, EDS was employed to confirm the successful deposition of nickel and cobalt oxides on the CNTF surface and to map their uniform distribution within the composite.

Figure 3a–e illustrates the elemental composition with their corresponding colors in the sample, verifying the existence of Ni, Co, O, and C in the provided spectrum. Furthermore, silicon detected in the sample is most likely attributable to the glass slide itself, which is usually composed of silica; likewise, carbon nanotube fiber fabricated on the glass slide contributes to the reason for the sample’s detection. The elemental abundance for the different elements has been shown in Figure 3f in which nickel cobalt and oxygen have the highest relative abundance with impurities including silicon, which is due to the glass substrate used to stick the CNTs fiber for the analysis purpose, while sodium, calcium, and potassium are the impurities associated with the precursors used for the fabrication of the microelectrode.

**Figure 3:**
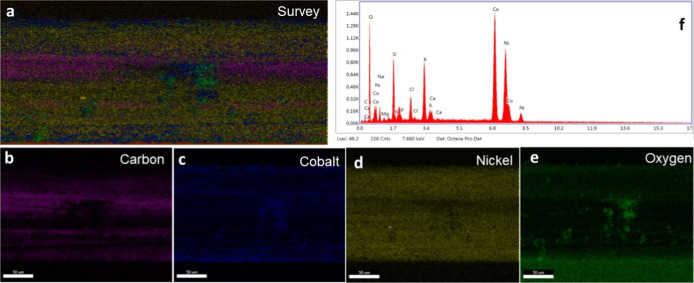
EDX elemental mapping for the NiCo–O modified CNTs fiber: (a) survey analysis; (b) carbon, (c) cobalt, (d) nickel, and (e) oxygen; and (f) elemental abundance.

#### 3.1.3 Raman and FTIR

Figure 4 represents the Raman spectra of carbon nanotubes (CNTs) and NiCo–O modified CNTs fiber which exhibited the typical prominent features such as the D band (∼1344 cm^–1^), G band (∼1572 cm^–1^), and the 2D band (∼2676 cm^–1^) associated with carbon nanotubes.^56^ The D band linked to the disorder or defects in the sp^2^ hybridized carbon lattice, while the G band reflects the in-plane vibration of sp^2^-bonded carbon atoms, indicative of graphitic order. After surface modification with nickel cobalt oxide (NiCo–O), the Raman spectrum showed characteristics peaks within the lower region of wavenumber at 528 cm^–1^ and 1064 cm^–1^, which are associated with the spherical structure of the NiCo–O phase and correspond to metal–oxygen vibrational modes.^57^

**Figure 4:**
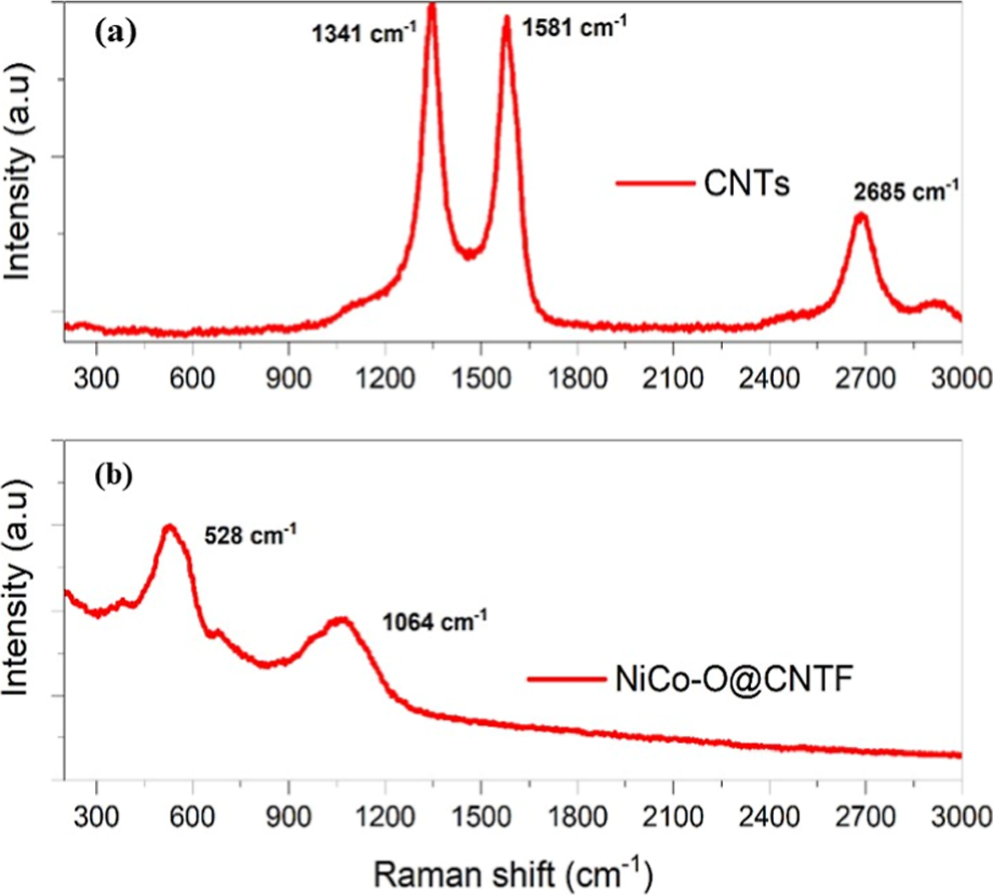
Raman spectra for the (a) bare CNTs and (b) modified CNTs fiber.

The FTIR spectra of pristine carbon nanotubes showed (Figure 5) the typical characteristic absorption bands around 3283 cm^–1^ corresponding to O–H stretching vibrations of adsorbed functional hydroxyl groups and a peak near 1730 cm^–1^ attributed to C=C stretching vibrations of the graphitic backbone. Additional bands appeared around 2921 cm^–1^ and 2857 cm^–1^ could be associated to the C–H stretching from residual organic impurities.^58^

**Figure 5:**
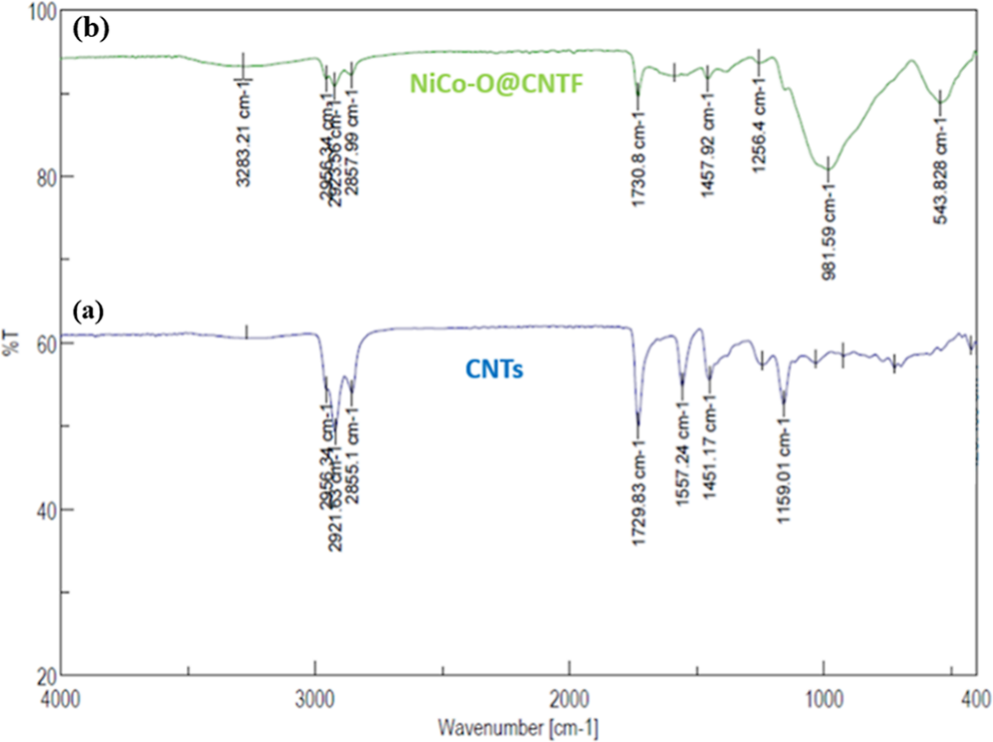
FTIR spectra of the (a) bare CNTs and (b) modified CNTs fiber.

Significant alterations are seen in the FTIR spectrum upon treatment with nickel cobalt oxide (NiCo–O@CNTF). It is confirmed that the metal oxide composite was successfully formed when additional bands appeared at 543 cm^–1^ with a wide band at 981 cm^–1^. These bands correspond to the metal–oxygen (M–O) stretching vibrations, notably Ni–O and Co–O bonds.^59^ Moreover, effective functionalization is indicated by a decrease in the intensity of the O–H and C–H bands, which might signify surface contact or partial metal oxide coating of the CNTs. All of these vibrational spectrum changes confirm that the composite (NiCo–O@CNTF) was formed and that there are robust interactions between the metal oxide nanoparticles and the CNT surface.

### 3.2 Electrocatalytic Activity Analysis at Different pH

The modified NiCo–O@CNTF fabricated electrode was evaluated through voltammetric analysis across a potential range of −0.4 to 0.8 V vs Ag/AgCl reference electrode. The electrochemical behavior of bare CNTF was investigated at pH 5.8 and 8.0, Figure 6a,b. In the absence of an analyte (buffer only), no redox peak was observed, indicating negligible intrinsic activity of the bare electrode. However, upon the addition of 1.25 mM AA under identical experimental conditions used for the modified electrode, a defined anodic peak appeared, confirming the direct oxidation of AA even on the bare CNTF, though with a significantly lower current response compared to the modified electrode.

**Figure 6:**
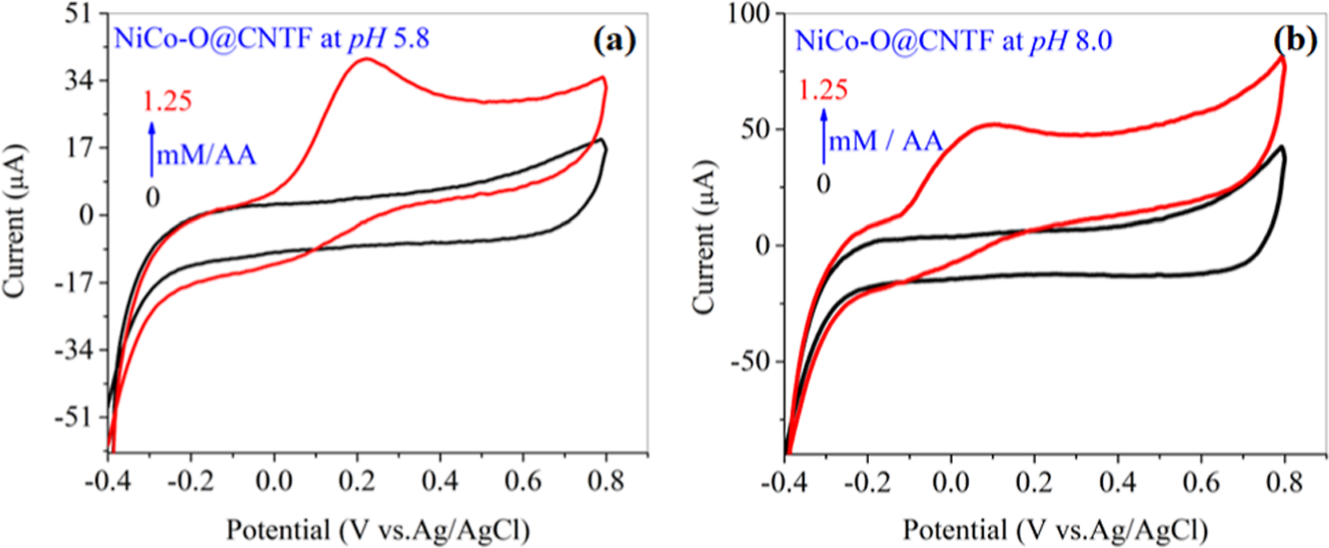
CV for the blank electrolyte and with analyte (AA) at the NiCo–O@CNTF electrode at (a) pH 5.8 and (b) pH 8.0.

Figure 7a shows a CV plot, taken at various concentrations ranging from 0.13 to 2.26 mM, revealing a noticeable increase at each concentration, indicating a clear rise in faradic current at pH 5.8 in 0.1 M PBS at a constant scan rate of 50 mV/s. The corresponding curve (Figure 7b) showed an excellent linear curve over the concentration range, resulting in an *R* ^2^ value of 0.99 within the required potential window. The influence of phosphate buffer on AA oxidation was also examined at pH 8.0 using the CV technique, as shown in Figure 7c, and its corresponding calibration curve with elevated sensitivity is presented in Figure 7d.

**Figure 7:**
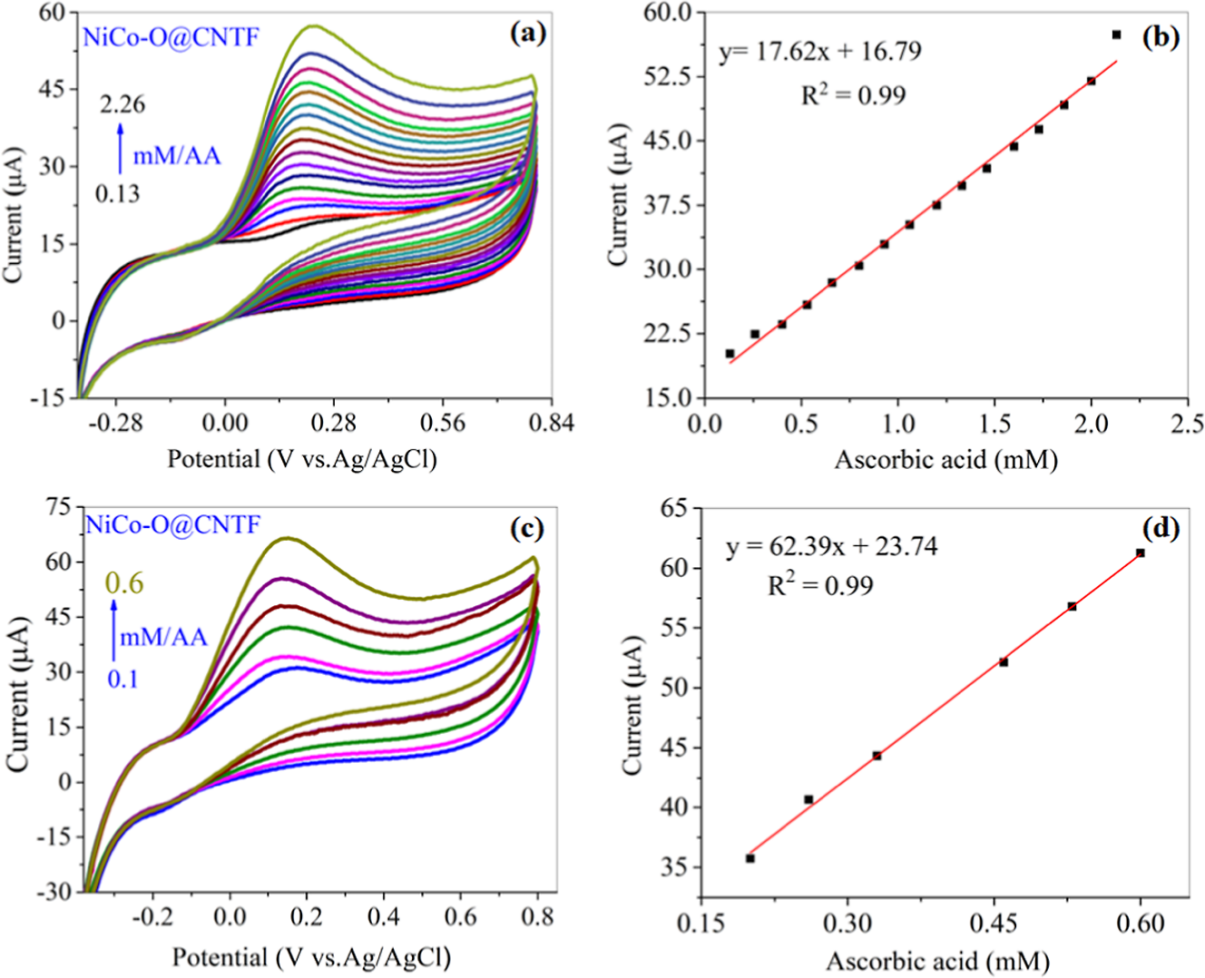
CV curves with increasing AA concentration at (a) pH 5.8 and (c) pH 8.0 with their (b, d) corresponding calibration plot at a fixed scan rate of 50 mV/s.

The anodic peak current attributed to ascorbic acid oxidation, initiated at ∼0.1 V and achieved a maximum at 0.2 V, with concentrations ranging from 0.1 to 0.6 mM, highlighting the pH conductivity optimizing the sensor performance. The comparative concentration graph displayed a corresponding linear connection with an *R* ^2^ value of 0.99, highlighting the consistent electrochemical behavior of the modified electrode.

To evaluate the influence of pH on the electrochemical oxidation of ascorbic acid (AA), an overlay cyclic voltammogram was recorded at a fixed concentration of 0.6 mM in two distinct pH media, acidic (pH 5.8) and basic (pH 8.0), as illustrated in Figure 8b. The anodic peak current exhibited a substantial enhancement under alkaline conditions, increasing from approximately 20 μA at pH 5.8 to nearly 65 μA at pH 8.0. This shift is indicative of increased ionization of ascorbic acid in basic media, preceding the formation of its deprotonated anionic form (ascorbate), which possesses greater electrochemical reactivity. The enhanced current response suggests improved charge transfer kinetics and increased availability of electron donors due to deprotonation at elevated pH. To further quantify this behavior, a bar graph comparison of electroanalytical performance across both pH conditions is presented in Figure 8a, integrating sensitivity, limit of detection (LOD), and limit of quantification (LOQ). The electrode demonstrated significantly higher sensitivity and lower detection thresholds under basic conditions. Figure 8c presents a detailed evaluation at pH 5.8, revealing diminished sensitivity and relatively higher LOD and LOQ values, consistent with the limited ionization and reduced conductivity at lower pH. In contrast, Figure 8d highlights the pronounced electrochemical performance at pH 8.0, where the LOD and LOQ are notably lower, affirming the electrode’s efficiency in alkaline environments. Collectively, these findings highlight the pH-responsive behavior of ascorbic acid and confirm the electrode’s capability to operate with higher accuracy and sensitivity at elevated pH levels. The comparison between overlay voltammograms and bar graph analyses reveals the correlation among ionization degree, conductivity, and signal output. This pH-modulated performance reflects the adaptive sensing nature of the modified electrode and its potential for reliable detection of AA in varying pH media.

**Figure 8:**
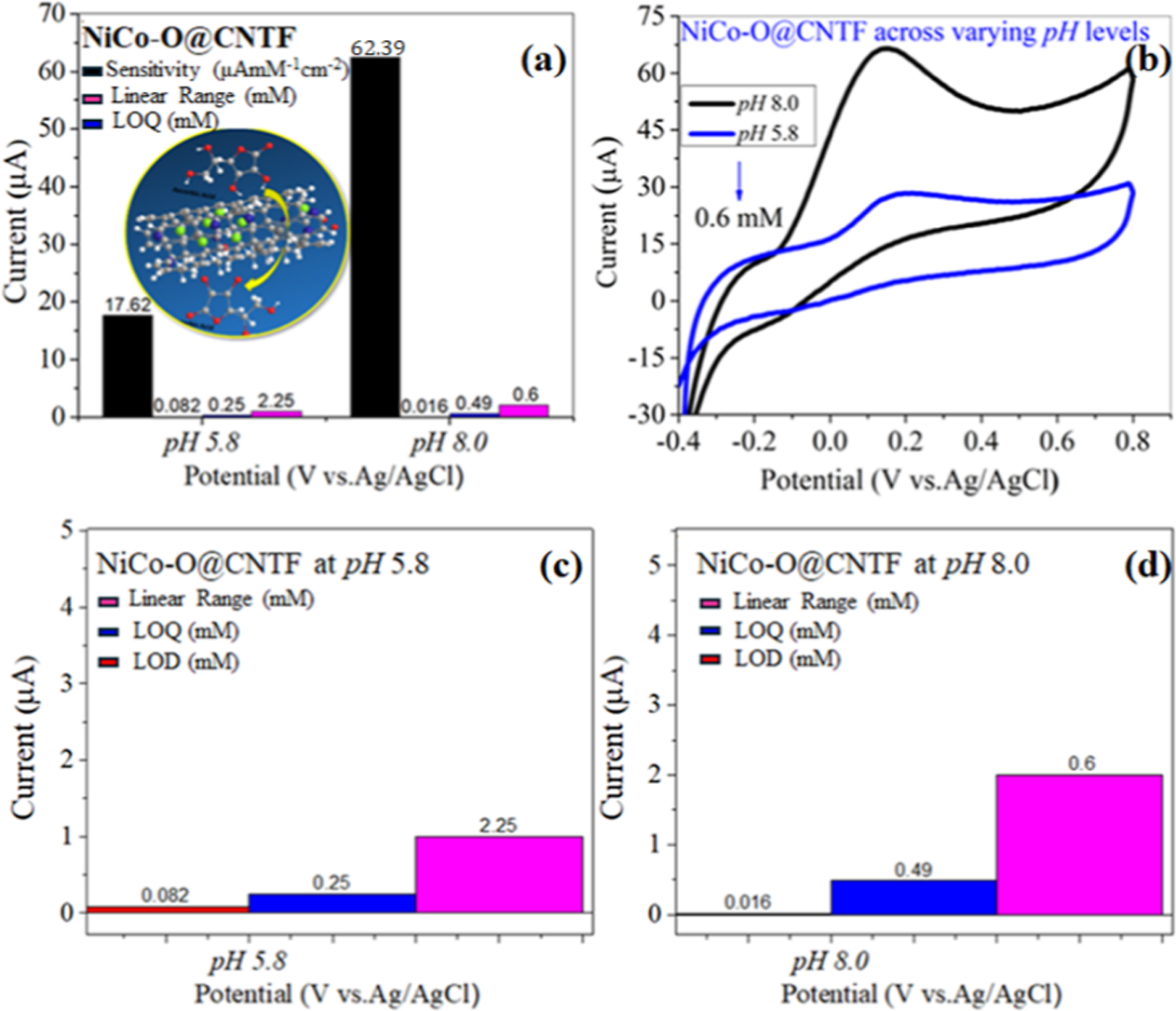
Bar graph for (a) comparison of electrochemical performance, overlay of (b) CVs with different pH, and (c, d) zoomed-in views of current at pH 5.8 and 8.0.

An overlay cyclic voltammogram (Figure 8b) was recorded for 0.6 mM ascorbic acid under both acidic (pH 5.8) and basic (pH 8.0) conditions to evaluate the effect of pH on the electrochemical performance. A marked enhancement in anodic peak current was observed at pH 8.0 (∼60 μA) compared to pH 5.8 (∼20 μA), indicating increased ionization of ascorbic acid in an alkaline medium. This ionization generates more charge carriers, thereby facilitating improved electron transfer and conductivity.

### 3.3 Effect of Scan Rate

To examine the electro-oxidation kinetics of AA at the modified electrode, a series of cyclic voltammograms were recorded at varied scan rates of 5–50 mV/s. The influence of the scan rate on the electrochemical oxidation of ascorbic acid was investigated at pH 5.8 and 8.0 on both bare CNTF and NiCo–O@CNTF electrodes (Figure 9a–f). For the bare CNTF, a gradual increase in the anodic current with scan rate was observed, reaching 1.58 μA at pH 5.8 (Figure 9a) and only 0.37 μA at pH 8.0 (Figure 9d), confirming its limited intrinsic catalytic activity. In contrast, the modified NiCo–O@CNTF displayed a significant enhancement in peak current with increasing scan rate, reaching 18.99 μA at pH 5.8 (Figure 9b) and 23.0 μA at pH 8.0 (Figure 9e), indicative of improved electron transfer kinetics at the modified surface independent of the potential window (−0.4 to 0.8 V). At pH 8.0, the conductivity was enhanced compared to pH 5.8 with the successive addition of 0.13 mM analyte (AA). This indicates that AA oxidation consistently occurs in a positive region, with overall electrochemical behavior staying identical across both pH levels. This rise in the peak current and the observed positive shift in the peak potential propose that the AA oxidation process is irreversible. The diffusion coefficient of the modified electrode (NiCo–O@CNTF) has been calculated as 6.24 × 10^–6^ cm^2^ s^–1^, which is far better compared to the diffusion coefficient of the bare carbon nanotubes-based electrode.

**Figure 9:**
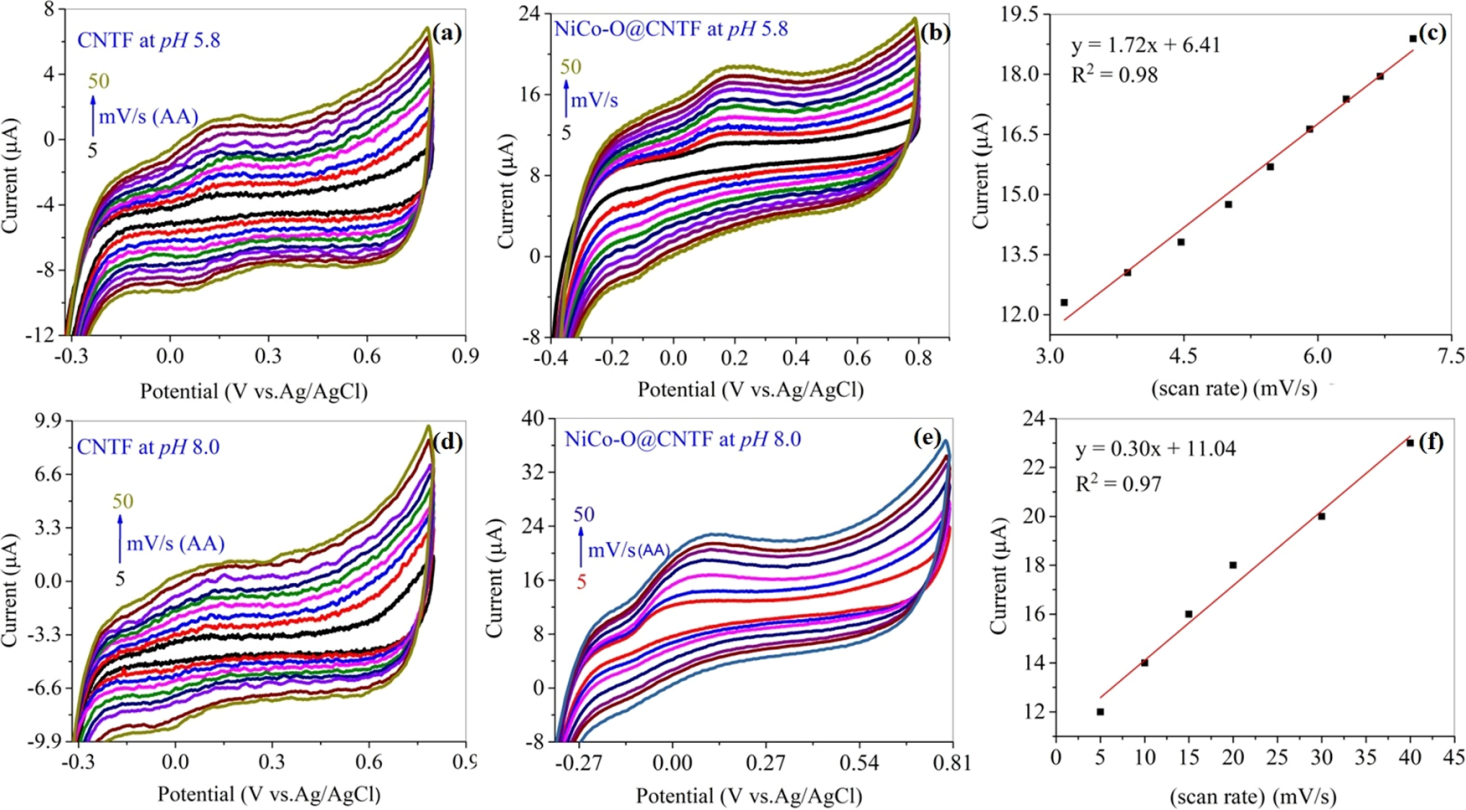
(a) Bare CNTF at pH 5.8. (b) NiCo–O@CNTF at pH 5.8. (c) Linear calibration at pH 5.8. (d) Bare CNTF at pH 8.0. (e) Modified at pH 8.0. (f) Linear calibration at pH 8.0.

The anodic peak current increases linearly with an increase in scan rate, depicted in Figure 9b,e. The linear relationship between anodic peak current (*i*_p_^a^) and the square root of scan rate (ν^1/2^) as represented by the regression equation (*I*_p_ = *m* × *v* ^1/2^ + *b*)^60^ at both pH levels (*R* ^2^ = 0.98 at pH 5.8, Figure 9c; *R* ^2^ = 0.97 at pH 8.0, Figure 9f) suggests that the oxidation of ascorbic acid is predominantly diffusion-controlled on the NiCo–O@CNTF surface. The higher current response at pH 8.0 further supports the enhanced catalytic activity in alkaline media, consistent with the pH-dependent deprotonation behavior discussed earlier.

### 3.4 EIS Studies

Electrochemical impedance spectroscopy (EIS) was employed to evaluate the charge transfer resistance (*R*_ct_) of the modified NiCo–O@CNTF electrode in comparison to the bare CNTs.^61^ The Nyquist plots (Figure 10) display a characteristic small arc in the high-frequency region, indicative of a charge-transfer-controlled process, whereas the linear segment in the low-frequency region corresponds to a diffusion-controlled process. In the high-frequency region, the *X*-axis intercept provides the equivalent series resistance (*R*_s_) of the electrode.

**Figure 10:**
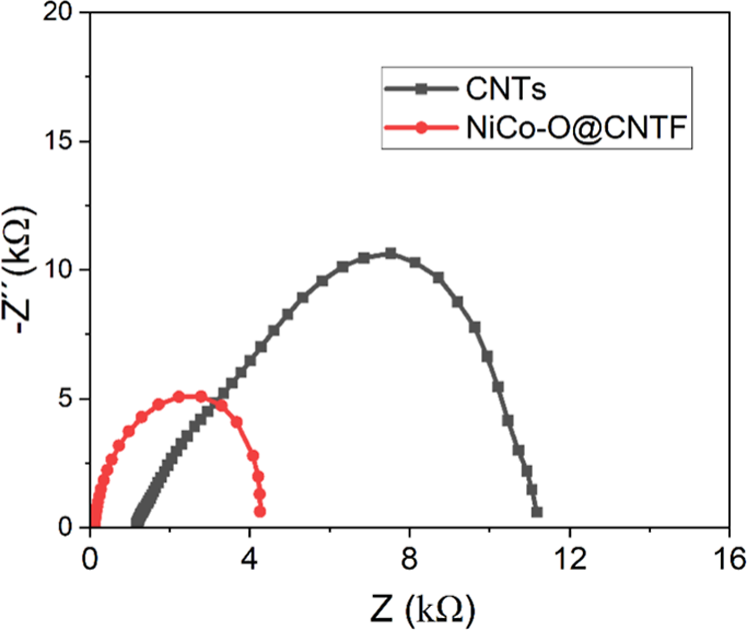
Electrochemical impedance spectra for the NiCo–O@CNTF and bare CNTs.

The semicircle in the high-frequency region represents an electron-transfer-limited process, with its diameter corresponding to *R*_ct_, reflecting the charge transfer kinetics at the electrode–electrolyte interface. Both bare and modified CNTs fiber electrodes exhibited the solution resistances of ∼0.17 kΩ and 1.1 kΩ, respectively. However, the *R*_ct_ value for NiCo–O@CNTF, estimated from the smaller semicircle, was ∼4.23 kΩ, significantly lower than that of the unmodified CNT fiber (∼9.43 kΩ). The reduced *R*_ct_ of the modified electrode (NiCo–O@CNTF) is consistent with the enhanced electron transfer characteristics inferred from the voltammetric and amperometric measurements.

### 3.5 Amperometric Response

At a potential window of 0.2 V, the chronoamperometric method was employed to assess the electrochemical performance of the fabricated electrode for the ascorbic acid (AA) detection (Figure 11a). The current response to AA oxidation increased linearly with the rise in concentration, demonstrating the electrode sensitivity, as shown in Figure 11b. Incorporating ascorbic acid significantly enhanced the current vs time response, yielding around 95% of the steady-state value. A current response was observed with the continuous addition of 0.13 mM AA every 30 s at a constant potential of 0.5 V vs Ag/AgCl in a solution containing a 0.1 M phosphate buffer electrolyte with continuous stirring. The successive addition of AA (Figure 11c) recorded an incremental staircase behavior. Figure 11d presents the linear calibration curve for ascorbic acid, exhibiting distinct current increments with an increasing concentration. The electrochemical response demonstrates excellent linearity (*R* ^2^ = 0.99) over the studied concentration range, confirming the electrode’s reliable sensing behavior. The strong correlation indicates that the approach is resilient and suitable under dynamic conditions.

**Figure 11:**
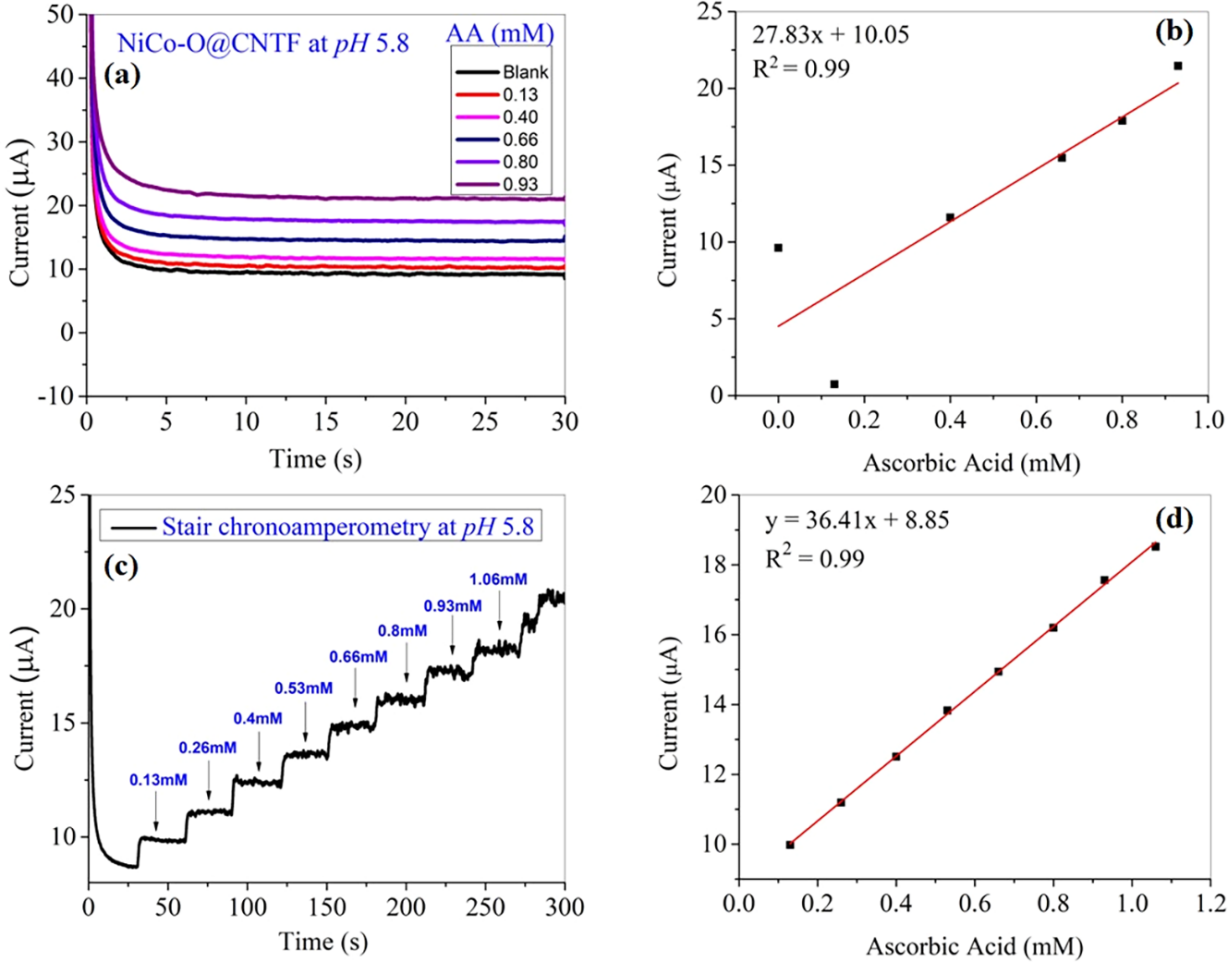
Straight chrono illustration with (a) different concentrations and its (b) linear calibration curve at pH 5.8 and (c) staircase chronoamperometry with the successive addition of AA at pH 5.8 and (d) its corresponding calibration curve.

A similar assessment at pH 8.0 (Figure 12a) was analyzed in a standard solution to examine the influence of the concentration on the anodic response. As the concentration of AA grew continuously from 0.06 mM to 0.40 mM, chronoamperometric measurements were taken at a constant potential. The chronoamperometric anodic current was recorded at varying concentrations of ascorbic acid (AA). The steady-state current values were plotted against the concentration, yielding a linear calibration curve with a high correlation coefficient (Figure 12b). The slope of the curve indicates the sensitivity of the electrode toward AA within the examined concentration range.

**Figure 12:**
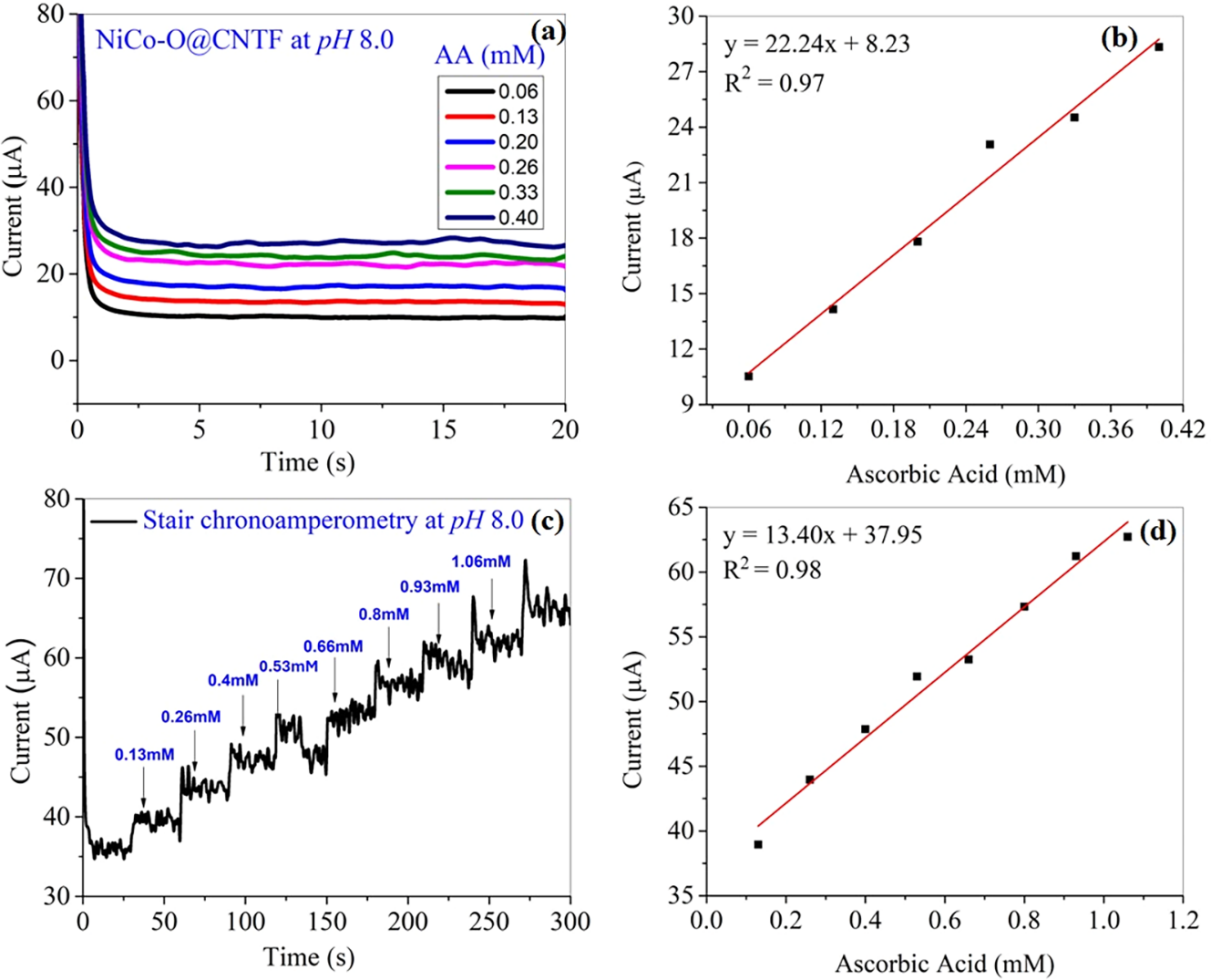
Straight chrono illustration with (a) different concentrations and its (b) linear calibration curve at pH 8.0 and (c) staircase chronoamperometry with the successive addition of AA at pH 8.0 and (d) its corresponding calibration curve.

Staircase chronoamperometry was performed for ascorbic acid (AA) in PBS of pH 8.0 with continuous stirring to assess the response over incremental additions. Every 30 s, AA was added in increments of 200 μL, creating clear and distinct stairs with each addition (Figure 12c). In Figure 12d, an anodic current responded significantly with each step and showed a linear relation over the concentration range, yielding an *R* ^2^ value of 0.98. The electrode’s sensitivity and detection limits vary with the pH values. The electrochemical performance of the NiCo–O@CNTF electrode was evaluated at pH levels of 5.8 and 8.0 to examine the influence of pH on the sensor response.

A comparative evaluation of recently reported AA sensors and the present work is summarized in Table 1. Most Ni- and Co-based electrodes, such as Ni–SnO_2_@Fabric and NiCo hexacyanoferrate (III)-GE/GCE, exhibit moderate sensitivities (<20 μA mM^–1^ cm^–2^) and linear ranges limited to neutral or mildly alkaline conditions. CoTSPc@MWCNT and CoV/MWCNTs offer slightly improved detection limits but lack pH-dependent optimization and mechanical flexibility. In contrast, the NiCo–O@CNTF microelectrode developed here combines a flexible fibrous framework with superior electrochemical performance, delivering the highest reported sensitivity (62.39 μA mM^–1^ cm^–2^ at pH 8.0) among Ni/Co-based systems, a rapid response time (5 s), and consistent performance in both near-neutral (pH 5.8) and alkaline (pH 8.0) media. This is a reasonable electrochemical sensing parameter as compared to the HPLC-based sensors, which showed an LOD of 4.83 μM.^67^ The unique CNTF-based structure enables easy integration into wearable electronics, making it a promising candidate for next-generation, portable, and real-time AA sensing platforms.

**Table 1:** Comparison of Reported Ni/Co-Based AA Sensors with the Present NiCo–O@CNTF Microelectrode in Terms of Sensitivity, Linear Range, and LOD

electrode	pH	Sensitivity (μA mM^–1^ cm^–2^)	Linear range (mM)	LOQ (mM)	LOD μM	ref
Ni–SnO2@fabric	-	-	0.1–0.6	0.057	19.11	^12^
CoTSPc@MWCNT	7.4	0.13	-	0.01	0.45	^62^
Ni NCs	7.0	-	0.001–3.212		0.1	^63^
NiCo hexacyanoferrate (III)-GE/GCE	8.0	-	0.2–2.0	-	0.15	^64^
CoV/MWCNTs	-	-	0.001–0.1		0.4	^65^
(Co-MOFs)@CNT	6.0	0.32	0.0005–0.06	-	0.16	^66^
NiCo–O@CNTF	5.8	17.62	0.13–2.25	0.25	82	this work
NiCo–O@CNTF	8.0	62.39	0.2–0.6	0.49	16	this work

### 3.6 Effect of pH

The influence of pH on the electrochemical oxidation of ascorbic acid was systematically investigated to determine the optimal conditions for signal enhancement. At acidic pH (5.8), ascorbic acid exists mainly in its protonated form, limiting electron transfer due to the reduced interaction with the Ni/Co active sites. With increasing pH, progressive deprotonation occurs, particularly at the hydroxyl group on carbon 3, yielding the ascorbate ion, which undergoes oxidation more readily. This deprotonation enhances electron transfer efficiency; however, at higher alkaline conditions (≥8.0), adsorbed hydroxyl species block active Ni/Co sites, limiting electron transfer and lowering the faradaic response.

The improved oxidation kinetics at elevated pH values are attributed to this pH-modulated deprotonation behavior, and the transition from protonated (ascorbic acid) to deprotonated (ascorbate ion) involves the loss of one proton (H^+^), as illustrated visually in Figure 13. Deprotonation provides the ascorbate ion (C_6_H_7_O_6_^–^) with a negative charge, making it more effective as an antioxidant by donating electrons and neutralizing free radicals. To visualize the pH-dependent electrochemical response of NiCo–O@CNTF, overlay cyclic voltammograms were recorded in the potential window of −0.4 to 0.8 V at pH 5.8, 6.5, 7.2, and 8.0 V (Figure 14). The CV profiles showed clear variations in peak positions and current intensities, indicating significant pH-driven changes in the interfacial electron-transfer kinetics.

**Figure 13:**
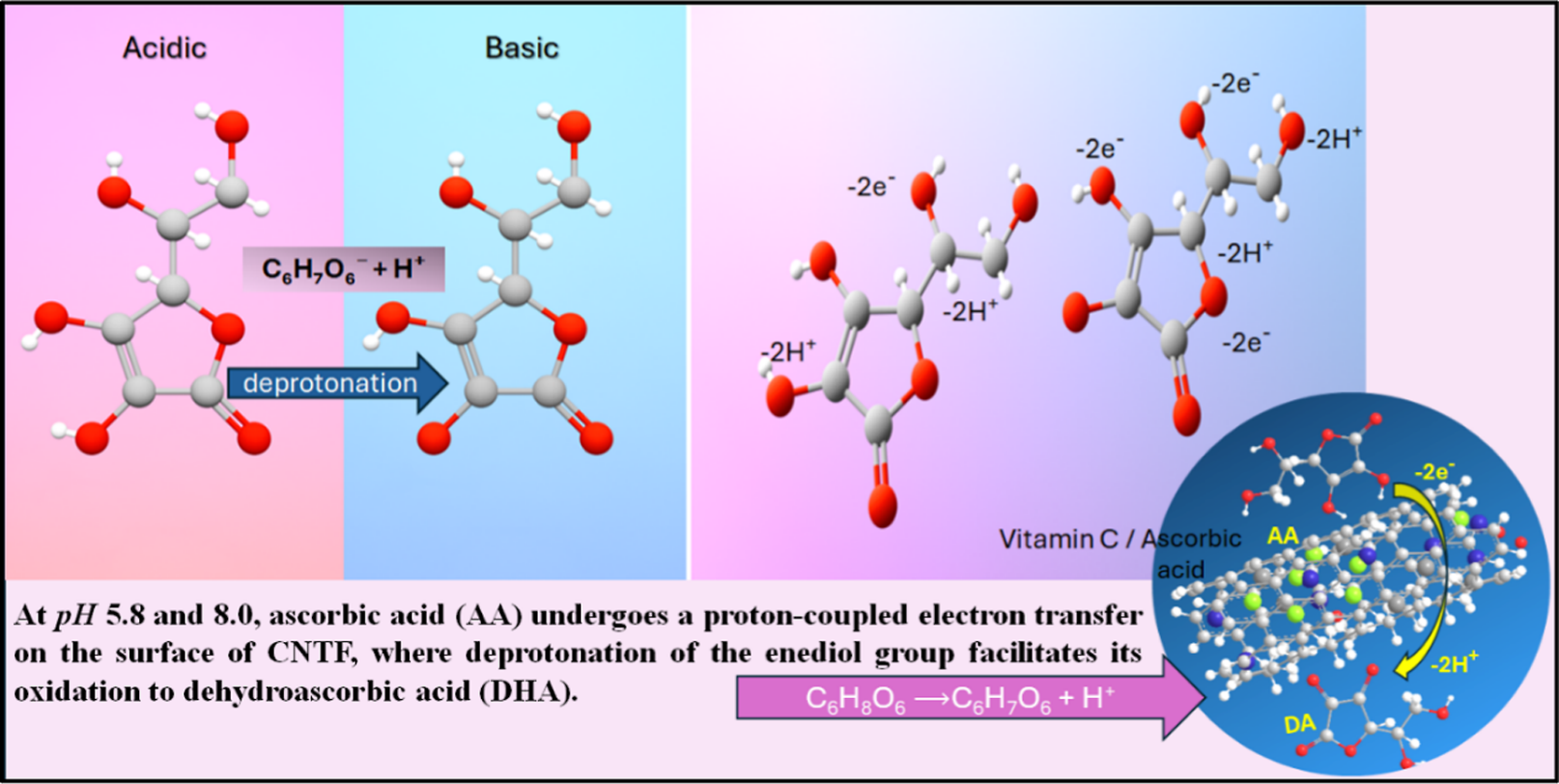
pH-dependent deprotonation mechanism of ascorbic acid to ascorbate ions at the modified electrode.

**Figure 14:**
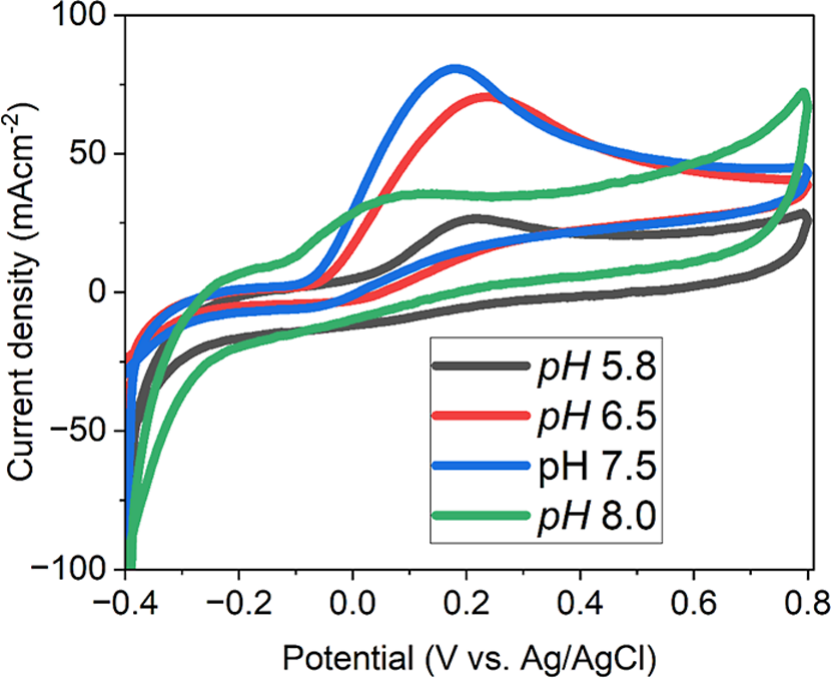
pH-dependent CVs of the modified electrode (NiCo–O@CNTF) at a fixed concentration and fixed SR of 50 mV/s.

The maximum anodic response was observed at pH 6.5 (0.23 V, ≈284.8 μA), followed by a slight decrease at pH 7.2 (0.17 V, ≈245.1 μA) and a sharp reduction at alkaline (pH 8.0, 0.0 V, ≈37.2 μA) and acidic (pH 5.8, 0.2 V, ≈28.8 μA) conditions. This nonlinear trend is consistent with previous NiCo-based reports,^64^ where the highest current near neutral pH was attributed to optimal AA deprotonation and strong electrostatic attraction to Ni/Co active sites. At lower pH, protonated AA species hinder electron transfer, whereas at high pH (≥8.0), surface hydroxyl adsorption and partial NiCo–O passivation suppress faradaic currents.

## 4 Conclusion

A flexible nonenzymatic microelectrode (NiCo–O@CNTF) was successfully fabricated via a simple and cost-effective electrodeposition approach. The sensor exhibited pronounced pH-dependent electrochemical behavior toward ascorbic acid, attributed to pH-modulated protonation–deprotonation kinetics. At pH 5.8, it achieved a sensitivity of 17.62 μA cm^–2^ mM^–1^, a linear range up to 2.25 mM, and a detection limit of 82 μM. In contrast, at pH 8.0, an obvious enhancement in anodic current was observed, with a higher sensitivity of 62.39 μA cm^–2^ mM^–1^ and a lower detection limit of 16 μM, demonstrating accelerated electron transfer under alkaline conditions. These findings highlight the potential of NiCo–O@CNTF as a flexible, low-cost, and efficient microelectrode platform for real-time vitamin C monitoring and future wearable sensing applications.
